# Supplementary material for: A Systematic Review of Extreme Phenotype Strategies to Search for Rare Variants in Genetic Studies of Complex Disorders
Source: Genes (Basel). 2020 Aug 25;11(9):987. doi: 10.3390/genes11090987 (PMC7564972; doi:10.3390/genes11090987)
Supplement: Supplementary file 1 [file genes-11-00987-s001.pdf]

genes

Table S1: Preferred Reporting Items for Systematic Reviews and Meta-Analyses (PRISMA)

Section/topic	#	Checklist item	Reported on page #
**TITLE**			
Title	1	Identify the report as a systematic review, meta-analysis, or both.	1
**ABSTRACT**			
Structured summary	2	Provide a structured summary including, as applicable: background; objectives; data sources; study eligibility criteria, participants, and interventions; study appraisal and synthesis methods; results; limitations; conclusions and implications of key findings; systematic review registration number.	1<br>*Registration number: N/A*
**INTRODUCTION**			
Rationale	3	Describe the rationale for the review in the context of what is already known.	1,2
Objectives	4	Provide an explicit statement of questions being addressed with reference to participants, interventions, comparisons, outcomes, and study design (PICOS).	2,3
**METHODS**			
Protocol and registration	5	Indicate if a review protocol exists, if and where it can be accessed (e.g., Web address), and, if available, provide registration information including registration number.	NA
Eligibility criteria	6	Specify study characteristics (e.g., PICOS, length of follow-up) and report characteristics (e.g., years considered, language, publication status) used as criteria for eligibility, giving rationale.	3,4
Information sources	7	Describe all information sources (e.g., databases with dates of coverage, contact with study authors to identify additional studies) in the search and date last searched.	3
Search	8	Present full electronic search strategy for at least one database, including any limits used, such that it could be repeated.	3
Study selection	9	State the process for selecting studies (i.e., screening, eligibility, included in systematic review, and, if applicable, included in the meta-analysis).	4
Data collection process	10	Describe method of data extraction from reports (e.g., piloted forms, independently, in duplicate) and any processes for obtaining and confirming data from investigators.	4,5
Data items	11	List and define all variables for which data were sought (e.g., PICOS, funding sources) and any assumptions and simplifications made.	4
Risk of bias in individual studies	12	Describe methods used for assessing risk of bias of individual studies (including specification of whether this was done at the study or outcome level) and how this information is to be used in any data synthesis.	5

Summary measures	13	State the principal summary measures (e.g., risk ratio, difference in means).	NA
Synthesis of results	14	Describe the methods of handling data and combining results of studies, if done, including measures of consistency (e.g., I^2) for each meta-analysis.	NA
Risk of bias across studies	15	Specify any assessment of risk of bias that may affect the cumulative evidence (e.g., publication bias, selective reporting within studies).	5
Additional analyses	16	Describe methods of additional analyses (e.g., sensitivity or subgroup analyses, meta-regression), if done, indicating which were pre-specified.	NA
**RESULTS**			
Study selection	17	Give numbers of studies screened, assessed for eligibility, and included in the review, with reasons for exclusions at each stage, ideally with a flow diagram.	5
Study characteristics	18	For each study, present characteristics for which data were extracted (e.g., study size, PICOS, follow-up period) and provide the citations.	5,6,7,8,9,10,11
Risk of bias within studies	19	Present data on risk of bias of each study and, if available, any outcome level assessment (see item 12).	5
Results of individual studies	20	For all outcomes considered (benefits or harms), present for each study: (a) simple summary data for each intervention group (b) effect estimates and confidence intervals, ideally with a forest plot.	5,6,7,8,9,10,11
Synthesis of results	21	Present results of each meta-analysis done, including confidence intervals and measures of consistency.	6
Risk of bias across studies	22	Present results of any assessment of risk of bias across studies (see Item 15).	5
Additional analysis	23	Give results of additional analyses, if done (e.g., sensitivity or subgroup analyses, meta-regression [see Item 16]).	NA
**DISCUSSION**			
Summary of evidence	24	Summarize the main findings including the strength of evidence for each main outcome; consider their relevance to key groups (e.g., healthcare providers, users, and policy makers).	12
Limitations	25	Discuss limitations at study and outcome level (e.g., risk of bias) and at review level (e.g., incomplete retrieval of identified research, reporting bias).	13
Conclusions	26	Provide a general interpretation of the results in the context of other evidence and implications for future research.	13
**FUNDING**			
Funding	27	Describe sources of funding for the systematic review and other support (e.g., supply of data); role of funders for the systematic review.	13

Table S2: Risk of bias of 19 EP studies

Reference	Study design	Sample size	Bias				
			Selection	Performance	Detection	Attrition	Reporting
Pullabhatla et al. (2017)	Family trios, Replication cohort	30 trios, *10995*	High	Low	High	Low	High
Johar et al.(2016)	Case-control, Cross-Sectional	47	Low	Low	Low	High	Low
Kunkle et al. (2017)	Case-control, Replication cohort	93, *8570*	High	Low	High	Low	High
Emond et al.(2012)	Case-control, Replication cohort	43, *696*	Low	Low	Low	Low	Low
Shtir et al. (2016)	Case-control, Cross-Sectional	43	Low	Low	Low	High	Low
Liu et al. (2016)	Case-control, Cross-Sectional	48 sporadic and 54 familial	Low	Low	Low	Low	Low
Husson et al.(2018)	Case-control, Cross-Sectional	92	Low	Low	Low	High	Low
Johar et al.(2015)	Case-control, Cross-Sectional	12	Low	Low	Low	Low	Low
Hiekkala et al.(2018)	Case report, Cross sectional	293	Low	Low	Low	High	Low
Qiao et al.(2018)	Case-control, Cross-Sectional	≈1769	High	Low	High	High	High
Bruse et al.(2016)	Case-control, Cross-Sectional	62	Low	Low	Low	Low	Low
Nuytemans et al.(2018)	Case report, Cross sectional	26(13 trios)	High	Low	High	High	High
Aubart et al.(2018)	Case-control, Cross sectional	51 EP and 8 sib-pairs	Low	Low	Low	High	Low
Gregson et al. (2018)	Case-control, Replication cohort	1258, *32965*	Low	Low	Low	Low	Low
Lee et al. (2018)	Case-control, Replication cohort	881, *274*	Low	Low	Low	Low	Low
Tomaiuolo et al. (2012)	Case-control, Replication cohort	1653, *909*	Low	Low	Low	High	Low
Goldberg-Stern et al. (2013)	Case-control, Cross sectional	14 familial cases	Low	Low	Low	High	Low
Shen et al. (2017)	Case-control, Cross sectional	884	Low	Low	Low	High	Low
Uzun et al. (2016)	Case report, Cross sectional	32	High	Low	High	High	High

Table S3: Quality assessment of EP studies

Reference	Inclusion & exclusion criteria								Percentage	Qualified
	Q1	Q2	Q3	Q4	Q5	Q6	Q7	Q8		
Kolek et al.(2014)	1	1	0	1	0	1	0	0	50	No
Amin et al .(2012)	1	1	1	1	0	1	0	1	75	No
Coassin et al. (2017)	1	1	1	1	0	1	0	1	75	No
Renaud et al.(2016)	1	1	0	1	1	1	0	0	62.5	No
Lee et al. (2018)	1	1	1	1	1	1	0	1	87.5	Yes
Charles et al. (2018)	1	1	1	1	0	1	0	1	75	No
Bjørnland et al. (2017)	1	1	1	1	0	1	1	1	87.5	No
Aubart et al.(2018)	1	1	1	1	1	1	0	1	87.5	Yes
Goldberg-Stern et al. (2013)	1	1	1	1	1	1	0	1	87.5	Yes
Pullabhatla et al. (2017)	1	1	1	0	1	1	0	1	75	Yes
Johar et al.(2016)	1	1	1	1	0	1	0	1	75	Yes
Kunkle et al. (2017)	1	1	1	1	1	1	0	1	87.5	Yes
Shen et al. (2017)	1	1	1	1	0	1	0	1	75	Yes
Emond et al.(2012)	1	1	1	0	1	1	0	1	75	Yes
Emond et al.(2015)	1	1	1	0	1	1	0	1	75	No
Shtir et al. (2016)	1	1	1	0	0	1	0	1	62.5	Yes
Liu et al. (2016)	1	1	1	1	1	1	0	1	87.5	Yes
Eerde et al. (2012)	1	1	1	1	0	1	0	1	75	No
Gregson et al. (2018)	1	1	1	1	0	1	0	1	75	Yes
Paternoster et al.(2011)	1	1	1	1	0	1	0	1	75	No
Husson et al.(2018)	1	1	1	1	1	1	0	1	87.5	Yes
Limou et al.(2010)	1	1	1	1	0	1	0	1	75	No
Johar et al.(2015)	1	1	1	1	1	1	0	1	87.5	Yes
Peloso et al. (2016)	1	1	1	1	0	1	0	1	75	No
Tomaiuolo et al. (2012)	1	1	1	1	0	1	1	1	87.5	Yes
Uzun et al. (2016)	1	1	1	1	0	1	0	1	75	Yes
Hiekkala et al.(2018)	1	1	1	1	1	1	0	1	87.5	Yes
Nuytemans et al.(2018)	1	1	1	1	0	1	0	1	75	Yes
Qiao et al.(2018)	1	1	1	1	1	1	0	1	87.5	Yes
Bruse et al.(2016)	1	1	1	1	0	1	0	1	75	Yes

Legend: 1= Yes, 0= No
